# Risk Factors of Early Otitis Media in the Danish National Birth Cohort

**DOI:** 10.1371/journal.pone.0166465

**Published:** 2016-11-16

**Authors:** Asbjørn Kørvel-Hanquist, Anders Koch, Janni Niclasen, Jesper Dammeye, Jørgen Lous, Sjurdur Frodi Olsen, Preben Homøe

**Affiliations:** 1 Department of Otorhinolaryngology and Maxillofacial Surgery, Zealand University Hospital, Køge, Denmark; 2 Department of Epidemiology Research, Statens Serum Institut, Copenhagen, Denmark; 3 Department of Infectious Disease Epidemiology, Statens Serum Institut, Copenhagen, Denmark; 4 Department of Psychology, University of Copenhagen, Copenhagen, Denmark; 5 Center for Collaborative Health, Aarhus University, Aarhus, Denmark; 6 Research Unit for General Practice Institute of Public Health, University of Southern Denmark, Odense, Denmark; 7 Department of Epidemiology Research, Centre for Fetal Programming, Statens Serum Institut, Copenhagen, Denmark; University of Southampton, UNITED KINGDOM

## Abstract

**Objective:**

To assess risk factors of otitis media (OM) in six-months-old children.

**Method:**

The sample consisted of 69,105 mothers and their children from the Danish National Birth Cohort. The women were interviewed twice during pregnancy and again 6 months after birth. The outcome “one or more” maternal reported episodes of OM at age six months. In total 37 factors were assessed, covering prenatal, maternal, perinatal and postnatal factors.

**Results:**

At age six months 5.3% (95% CI 5.1–5.5) of the children had experienced one or more episodes of OM. From the regression analysis, 11 variables were associated with a risk of OM. When a Bonferroni correction was introduced, gender, prematurity, parity, maternal age, maternal self-estimated health, taking penicillin during pregnancy, and terminating breastfeeding before age six months, was associated with a risk of early OM. The adjusted ORs of OM for boys versus girls was 1.30 (95% CI 1.18–1.44). The OR having one sibling versus no siblings was 3.0 (95% CI 2.64–3.41). If the woman had been taking penicillin during pregnancy, the OR was 1.35 (95% CI 1.15–1.58). Children born before 38^th^ gestational week had an increased OR for early OM of 1.49 (95% CI 1.21–1.82). Children of young women had an increased OR of early OM compared to children of older women. Additionally, children of women who rated their own health low compared to those rating their health as high, had an increased OR of 1.38 (95% CI 1.10–1.74). Finally, children being breastfeed less than 6 months, had an increased OR of 1.42 (95% CI 1.28–1.58) compared to children being breastfeed beyond 6 months.

**Conclusion:**

These findings indicate that prenatal factors are of less importance regarding early OM before the age of six months. Postnatal risk factors seem to pose the main risk of early OM.

## Introduction

Otitis media (OM) is one of the most common infections in early childhood and may be regarded as a complication of upper respiratory tract infections[1]. OM covers a diverse group of diseases, which contain some degree of inflammation or infection located in the middle ear and the tympanic membrane. Also, OM is the primary cause of narrow and broad spectrum antibiotic prescriptions for children in western countries[2,3]. At introduction of the conjugate pneumococcus vaccination the incidence of OM was expected to decline, however the decline in otitis media due to introduction of the pneumococcal vaccination has only been modest, in addition no decline in prescription of antibiotics has been observed, at least not in Denmark[4,5]. It is estimated that 60–70% of all children will experience one or more episodes of OM during childhood, and approximately 24% will experience more than 3 episodes of OM during childhood [6,7]. As a consequence, OM remains a significant childhood disease and can thus be considered a substantial ecological and economical health burden[8–10].

In an attempt to assess child susceptibility to recurrent episodes of OM, studies have found early-onset OM to be an important risk factor for recurrent otitis media(ROM)[11–16]. Studies indicate that recurrent childhood OM have prolonged effects on hearing, speech development, behavior and school performance [17–23]. Successful delay in onset of the first episode of OM could therefore decrease the frequency of ROM and later health and developmental consequences due to early OM and ROM.

Few studies have evaluated prenatal risk factors. So far, a small number of studies have reported significant associations between smoking during pregnancy, alcohol consumption during pregnancy, gestational use of medication, maternal exposure to dioxin-like compounds, and exposure to organochlorines and early OM or ROM [7,24–29]. Many existing studies however, are not conclusive regarding which prenatal and environmental factors contributes to increased risk of early OM. Previous studies may suffer from insufficient sample size and lack of some relevant variables [30,31].

The objectives of the present study were to examine environmental risk factors of early OM in offspring using data from a large national cohort. These environmental risk factors included socioeconomic factors, dietary habits, smoking, alcohol consumption, disease during pregnancy and use of medicine during pregnancy. Peri- and postnatal factors have also been taken into account.

## Materials and Methods

The present study was based on data from the Danish National Birth Cohort (DNBC) collecting baseline data between 1996 and 2002. The women were recruited at their first antenatal visit with their GP. When the women were enrolled they were offered four interviews, two prenatally at approximately weeks 12 and 30, and twice after delivery at age six and 18 months of age of their child[32].

In this study it was not possible to differentiate between different kinds of early OM as it depends on parent-reported outcome measures. Physicians often do not distinguish between different infections in the middle ear, hence it was not expected that parents were able to separate one kind of middle ear infection from another.

### Settings

The DNBC is a national multipurpose birth cohort. Approximately 60% of the invited pregnant women accepted participation. A total of 100,329 pregnancies were enrolled in the cohort equivalent to a participation rate of 30% of all eligible Danish pregnant women [33]. The same woman could be represented with more than one pregnancy, if she had multiple pregnancies during the enrollment period. The cohort has been described in more details elsewhere[32].

The DNBC has been approved by the Committee on Biomedical Research Ethics (KF) 01-471/94.

### Exclusion/Inclusion parameters

**Fig 1:**
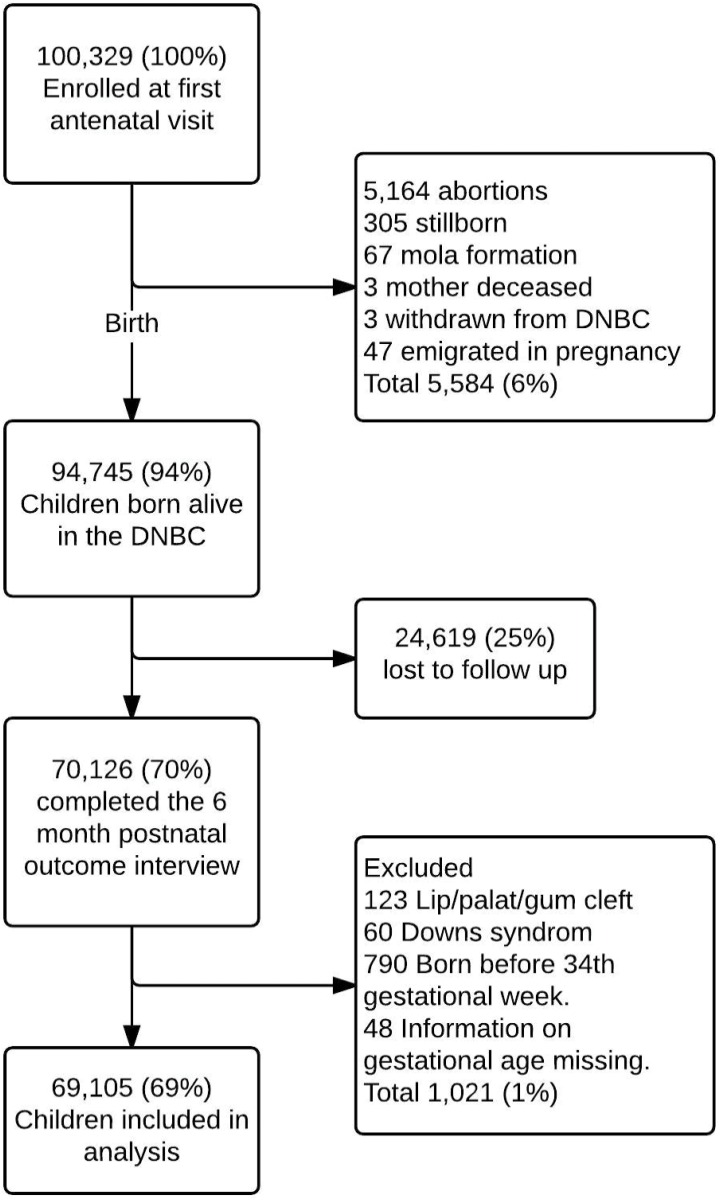
Flowchart of subject participation, loss to follow-up and exclusion in the Danish National Birth Cohort.

A complete case analysis was carried out, in which all participants with full information on OM from the third interview (six months of age) were included. Exclusion criteria were: children born premature before gestational week 34 because of increased incidence of infections[34–36]; children with lip-palate malformation due to cranial deformation resulting in increased OM incidence[37], and Down syndrome (Fig 1).

#### Exposure variables

Based on the literature, 37 variables were selected for the analyses and risk estimations, including pre- and postnatal risk factors. The variables were selected, if they were suspected to influence the health of the newborn child through epigenetic changes, immunological changes or if they were known to influence the risk of OM. Some were not environmental factors, but were included because they were important for adjustment in the multiple logistic risk estimates.

#### Outcome variables

The primary outcome was maternal-reported episodes of OM within the first six months. In the analyses, the outcomes were dichotomized. The outcome groups were “no OM” and “1 or more episodes of OM” at age six months of the child. If the woman had completed the interview, but items from the interview regarding OM were missing or otherwise inconclusive, the woman and her child were defined as having missing data and included in the analyses as “missing”.

### Statistical methods

All analyses were performed using SAS 9.4 software (SAS institute Inc. Cary, North Carolina). We performed Chi-square tests to examine the background characteristics of already known and potential risk factors for the “no OM” and the “1 or more OM” group. Significance level for the Chi-square test was set at the 5% level. To estimate an adjusted odds ratio, a multiple logistic regression analysis was performed. Bonferroni correction was used to estimate an adjusted p-value for the multiple logistic model, to counteract the effect of multiple testing.

To make the logistic model we firstly tested related variables for internal correlation. Variables were grouped with related variables in an attempt to find the most important exposures and possible latent variables (Table 1). Each group of variables was tested against the outcome in a multiple logistic regression model. If an exposure variable was found to be significantly associated with the outcome in the logistic model with a group of related variables, it was included in the final multiple logistic regression model as an independent variable. The final model included 22 different exposure variables. To test the strength of the results, descriptive statistics at baseline for those attending complete follow-up compared to those not-attending follow-up were made using Chi-square test.

**Table 1:** Chosen exposure variables shown in groups for internal correlation analyses.

Factors assessed	
Socioeconomic status	*
Maternal age	*
Single parenthood	
Maternal smoking during pregnancy	
Parental smoking after births	
Maternal alcohol intake during pregnancy	
Maternal self-reported health status	*
Termination of breastfeeding	*
Singleton or multiple birth	
Birth weight	*
Gestational age of birth (before week 38)	*
Way of birth	
Parity	*
Housing size	
Daycare center attendance first 6 month	*
Vegetarian diet during pregnancy	*
Iron supplement during pregnancy	*
Anaemia during pregnancy	*
Maternal asthma	
Maternal allergy	*
Taking asthma medicine during pregnancy	
Taking allergy medicine during pregnancy	
Infection during pregnancy	*
Taking non-penicillin antibiotics during pregnancy	*
Taking penicillin during pregnancy	*
Taking paracetamol during pregnancy	
Taking non-steroid anti-inflammatory drugs during pregnancy	
Taking acetylsalicylic acid during pregnancy	*
Tea consumption during pregnancy	*
Coffee consumption during pregnancy	*
Cola consumption during pregnancy	*
Diabetes during pregnancy	
Exercise during pregnancy	*
Weight status before pregnancy based on BMI	*
Weight change during pregnancy	

* Risk factors found to be significantly associated to early otitis media, when tested in a multiple logistic regression model with variables in the group and included in adjusted model.

## Results

Of the cohort of 100,329 pregnancies, a total of 94,745 (94.4%) pregnancies resulted in the birth of a live born child. Between birth and the first postnatal interview 24,619 (24.5%) were lost during follow-up. Only three women formally withdrew from the cohort, the rest were non-responders on the exposure items or the item regarding OM. We excluded 973 (1.0%) from the study cohort due to the exclusion criteria. Final cohort for analysis included a total of 69,105 pregnancies (68.9%) of the total cohort (Fig 1).

At age six months 5.3% (95% confidence interval 5.1% - 5.5%) of the children had suffered from at least one episode of early OM. A number of variables were unequally distributed between the “no OM” and the “1 or more OM” groups. These variables (displayed in Table 1) were tested for internal-correlation, with the exception of gender that was included without being tested for internal correlation. Only the variables singleton versus plural birth, paracetamol, vitamins, fish-oil and being vaccinated were not associated with change in risk of early OM in the crude univariate analysis (Table 2).

**Table 2:** Prevalence, characteristics and crude odds ratio (OR) of exposure for early otitis media within first 6 months in the Danish National Birth Cohort.

Risk factor analysis	Analysis/missing	No early OM	≥1 early OM	Crude	
N = 69.105				OR (95% CI)	P-value
**Gender** °	69062/43				<0.0001
Boy		33131	2128	1.65 (1.27–1.45)	
Girl		32275	1528	ref	
**Way of birth** °	69067/38				0.0280
Natural birth		55793	3070	ref	
Caesarean section		9618	586	1.11 (1.01–1.21)	
**Birth weight** °	67183/1922				
Very low birth weight (< 1500 g)		38	6	3.00 (1.27–7.10)	<0.0001
Low birth weight (1500 g—2499 g)		1179	62	1.00 (0.77–1.29)	
Normal birth weight (2500 g—4000 g)		48655	2569	ref	
Weight = > 4000 grams		13751	923	1.27 (1.18–1.37)	
**Gestational age at birth** ^3^	51212/17893				0.0002
Preterm (Before 38th gestational week)		2732	183	1.34 (1.15–1.57)	
Term (in or after 38th gestational week)		46003	2294	ref	
**Singleton or multiple birth** °	69105/0				
Singleton		65436	3657	ref	0.4128
Multiple birth		12	0	-	
**Parity** °	69102/3				<0.0001
1		31499	940	ref	
2		23638	1869	2.65 (2.45–2.87)	
3		8423	705	2.81 (2.54–3.10)	
4+		1885	143	2.54 (2.12–3.05)	
**Social class** °	66219/2886				<0.0001
1		13084	626	0.78 (0.71–0.86)	
2		20221	1057	0.85 (0.79–0.93)	
3		23816	1457	ref	
4		4346	306	1.15 (1.01–1.31)	
5		1220	86	1.15 (0.92–1.44)	
**Maternal age at birth** °	68595/510				0.0121
Age < 20		298	12	0.76 (0.43–1.36)	
20 < = age < 30		30831	1636	ref	
30 < = age <40		33219	1956	1.11 (1.04–1.19)	
40 < = age		613	30	0.92 (0.64–1.34)	
**Single parenthood** ^3^	69037/68				0.6419
No		63967	3577	ref	
Yes		1418	75	0.95 (0.75–1.20)	
**Housing conditions** ^1^	44086/25019				<0.0001
Under 100 square meter		23227	1057	ref	
Between 100 and 150 square meter		16220	1013	1.37 (1.26–1.50)	
Over 150 square meter		2430	139	1.26 (1.05–1.51)	
**Maternal smoking during pregnancy** ^2^	65446/3659				<0.0001
Non-smoking		52443	2827	ref	
Between 1 and 10 cigarettes a day		6948	462	1.23 (1.12–1.37)	
More than 10 cigarettes a day		2593	173	1.24 (1.06–1.45)	
**Maternal alcohol during pregnancy** ^2^	65305/3800				0.0450
No alcohol		42600	2438	ref	
Yes. 2 or less units a week		15197	784	0.90 (0.83–0.98)	
Yes. more than 2 units a week		4054	232	1.00 (0.87–1.15)	
**Vegetarian** ^2^	65441/3664				0.0059
No		61228	3436	ref	
Yes		753	24	0.57 (0.38–0.85)	
**Tea consumption during pregnancy** ^2^	54635/14470				<0.0001
No		24358	1499	ref	
Yes. > 1 ≤ 5 cups daily		23160	1200	0.84 (0.78–0.91)	
Yes. > 5 cups daily		4215	203	0.78 (0.67–0.91)	
**Coffee consumption during pregnancy** ^2^	58586/10519				0.0023
No		30512	1653	ref	
Yes. > 1 ≤ 5 cups daily		21248	1277	1.11 (1.03–1.20)	
Yes. > 5 cups daily		3655	241	1.22 (1.06–1.40)	
**Cola consumption during pregnancy** ^2^	65461/3644				<0.0001
No		20096	1023	ref	
< 1 liter a week		30782	1727	1.10 (1.02–1.19)	
≥ 1 liters a week		11124	709	1.25 (1.14–1.38)	
**Taking fish oil during pregnancy** ^2^ ^3^	65958/3147				0.2487
No		57847	3256	ref	
Yes		4615	240	0.92 (0.81–1.06)	
**Taking vitamins during pregnancy** ^2^ ^3^	68549/556				0.2140
No		4270	257	ref	
Yes		60661	3361	0.92 (0.81–1.05)	
**Taking iron supplement during pregnancy** ^2^ ^3^	68147/958				0.0004
No		9860	630	1.17 (1.07–1.28)	
Yes		54679	2978	ref	
**Anemia during pregnancy** ^2^	65343/3762				0.0136
No		57271	3155	ref	
Yes		4620	297	1.17 (1.03–1.32)	
**Infections during pregnancy** ^2^	65458/3647				<0.0001
No		54990	2977	ref	
Yes		7006	485	1.28 (1.16–1.41)	
**Diabetes during pregnancy** ^2^ ^3^	65248/3857				0.0086
No		60784	3372	ref	
Yes		1015	77	1.37 (1.08–1.73)	
**Maternal asthma ever and still suffer from it** ^1^	66087/3018				0.0013
No		59326	3291	ref	
Yes		3244	226	1.26 (1.09–1.44)	
**Maternal allergy ever** ^1^	66312/2793				<0.0001
No		43326	2308	ref	
Yes		19451	1227	1.18 (1.10–1.27)	
**Exercise during pregnancy** ^2^	65775/3330				<0.0001
No		42917	2563	ref	
Yes		19373	922	0.80 (0.74–0.86)	
**Weight status before pregnancy based on BMI** ^1^	69105/0				<0.0001
Underweight		6225	306	0.94 (0.83–1.06)	
Normal weight		42001	2204	ref	
Overweight		12076	755	1.19 (1.09–1.30)	
Obese		5146	392	1.45 (1.30–1.62)	
**Weight change during pregnancy** ^3^	68473/632				<0.0001
Weight loss		467	48	1.90 (1.40–2.56)	
No weight loss or less than 10 kg weight gain		12626	770	1.12 (1.04–1.22)	
Weight gain > 10 kg		51754	2808	ref	
**Maternal self-estimated health** ^1^	66433/2672				<0.0001
Below average		2190	184	1.66 (1.42–1.92)	
Average		27548	1680	1.20 (1.12–1.29)	
Above average		33152	1679	ref	
**Vaccinated during pregnancy** ^2^	65158/3947				0.3269
No		61176	3433	ref	
Yes		525	24	0.81 (0.54–1.23)	
**Taking asthma medicine during pregnancy** ^3^	69034/71				0.0026
No		63728	3530	ref	
Yes		1654	122	1.33 (1.10–1.61)	
**Taking allergy medicine during pregnancy** ^3^	69030/75				0.0120
No		63774	3535	ref	
Yes		1607	114	1.28 (1.05–1.55)	
**Taking paracetamol during pregnancy** ^2^	65775/3330				0.5244
No		5277	306	ref	
Yes		57013	3179	0.96 (0.85–1.08)	
**Taking NSAID during pregnancy** ^2^	65775/3330				<0.0001
No		31744	2004	ref	
Yes		30546	1481	0.77 (0.72–0.82)	
**Taking acetylsalicylic acid during pregnancy** ^2^	65775/3330				<0.0001
No		32317	2044	ref	
Yes		29973	1441	0.76 (0.71–0.81)	
**Taking non-penicillin antibiotics during pregnancy** ^2^	65370/3735				0.0021
No		56744	3113	ref	
Yes		5173	340	1.20 (1.07–1.34)	
**Taking penicillin during pregnancy** ^2^	65508/3597				<0.0001
No penicillin		56337	3026	ref	
Penicillin		5708	437	1.42 (1.27–1.58)	
**Termination of breastfeeding** ^3^	69104/1				<0.0001
Before 6 months of age		23738	1629	1.41 (1.32–1.51)	
After 6 months of age		41709	2028	ref	
**Daycare center during first 6 month** ^3^	69012/93				<0.0001
No daycare center attendance		64508	3561	ref	
Some daycare center attendance		938	5	1.83 (1.48–2.27)	
**Parental smoking after births** ^3^	69101/4				<0.0001
Non-smoking		58749	3195	ref	
Daily smoking (> 1 cigarette a day)		6695	462	1.27 (1.15–1.40)	

° Data from the statistics Denmark.

^1^ Data the from first telephone interview at gestational week 12.

^2^ Data from the second telephone interview at gestational week 30.

^3^ Data from the third interview at 6 month post partum.

From the adjusted multiple logistic model we found 11 variables to be associated with increased or decreased risk of early OM (Table 3). Prenatal and maternal characteristics associated with increased or decreased risk of early OM were gender of the child, gestational age at birth, maternal age at birth, if the woman ever had suffered from allergy, weight status before pregnancy, maternal self-estimated health, and use of antibiotics during pregnancy. Taking antibiotics other than penicillin during the period from the 12^th^ until the 30^th^ gestational week was significantly associated with an increased risk of early OM in the adjusted model but not significant after Bonferroni correction. As opposed to this, maternal report of having taken penicillin during the same period from the 12^th^ until the 30^th^ gestational week was significantly related to an increased risk of early OM with odds ratio (OR) at 1.35 (95% confidence interval 1.15–1.58) in the adjusted model corrected with Bonferroni. This would result in a population attributable fraction of 112 children, which could avoid having early OM equal to 3.2% of the early OM cases, if the exposure were removed from the population. The effect on early OM risk from intake of coffee and cola from the crude analysis, were not significant in the adjusted risk estimate. Tea consumption was associated with a decreased risk in the crude analysis. In the adjusted model, only the analysis for trend in the consumption of tea was still significant. In addition, taking iron supplement during the last part of pregnancy tended towards a decreased risk of early OM, however this was also insignificant in the adjusted model.

**Table 3:** Adjusted odds ratio (OR) for otitis media within the first 6 months in the Danish National Birth Cohort.

Risk factor analysis	OR	(95% CI)	Adjusted	P-value	
N = 69.105				Trend	Bonferroni (n = 22)
**Gender** °			<0.0001	-	<0.005
Boy	1.30	(1.18–1.44)			
Girl	ref				
**Birth weight** °			0.3179	0.0867	0.86*
Very low birth weight (< 1500 g)	-	-**			
Low birth weight (1500 g—2499 g)	0.75	(0.47–1.18)			
Normal birth weight (2500 g—4000 g)	ref				
Weight = > 4000 grams	1.08	(0.97–1.22)			
**Gestational age at birth** ^3^			0.0001	-	<0.005
Preterm (Before 38th gestational week)	1.49	(1.21–1.82)			
Term (in or after 38th gestational week)	ref				
**Parity** °			<0.0001	<0.0001	<0.005*
1	ref				
2	3.00	(2.64–3.41)			
3	3.65	(3.09–4.30)			
4+	3.88	(2.97–5.09)			
**Social class** °			0.5567	0.5973	1.00*
1	1.01	(0.88–1.17)			
2	0.93	(0.82–1.05)			
3	ref				
4	0.93	(0.76–1.12)			
5	0.84	(0.60–1.18)			
**Maternal age at birth** °			0.0003	<0.0001	<0.005*
Age < 20	1.45	(0.67–3.14)			
20 < = age < 30	ref				
30 < = age <40	0.79	(0.70–0.88)			
40 < = age	0.72				
**Vegetarian**^2^			0.1338	-	0.96
No	ref				
Yes	0.62	(0.33–1.61)			
**Tea during pregnancy** ^2^			0.0789	0.0355	0.55*
No	1.14	(0.94–1.38)			
Yes, > 1 ≤ 5 cups daily	1.02	(0.84–1.23)			
Yes, > 5 cups daily	ref				
**Coffee during pregnancy** ^2^			0.9191	0.8333	1.00*
No	ref				
Yes, > 1 ≤ 5 cups daily	1.00	(0.89–1.11)			
Yes, > 5 cups daily	1.04	(0.86–1.25)			
**Cola during pregnancy** ^2^			0.8846	0.7765	1.00*
No	ref				
< 1 liter a week	1.01	(0.91–1.13)			
> = 1 liters a week	0.98	(0.84–1.13)			
**Taking iron supplement during pregnancy** ^2^ ^3^			0.1291	-	0.95
No	0.90	(0.78–1.03)			
Yes	ref				
**Anemia during pregnancy** ^2^			0.1803	-	0.99
No	ref				
Yes	1.14	(0.94–1.38)			
**Infections during pregnancy** ^2^			0.9116	-	1.00
No	ref				
Yes	1.01	(0.86–1.18)			
**Maternal allergy ever** ^1^			0.0100	-	0.20
No	ref				
Yes	1.15	(1.03–1.28)			
**Exercise during pregnancy** ^2^			0.3092	-	1.00
No	ref				
Yes	1.06	(0.95–1.19)			
**Weight status before pregnancy based on BMI** ^1^			0.0279	0.0119	0.23*
Underweight	0.88	(0.70–1.11)			
Normal weight	ref				
Overweight	1.00	(0.88–1.14)			
Obese	1.24	(1.06–1.46)			
**Maternal self-estimated health** ^1^			0.0060	0.0019	0.04*
Below average	1.38	(1.10–1.74)			
Average	1.13	(1.02–1.25)			
Above average	ref				
**Taking acetylsalicylic acid during pregnancy** ^2^			0.0751	-	0.82
No	ref				
Yes	0.91	(0.82–1.01)			
**Taking non-penicillin antibiotics during pregnancy** ^2^			0.0491	-	0.67
No	ref				
Yes	1.20	(1.00–1.43)			
**Taking penicillin during pregnancy** ^2^			0.0003	-	0.01
No penicillin	ref				
Penicillin	1.35	(1.15–1.58)			
**Termination of breastfeeding** ^3^			<0.0001	-	<0.005
Before 6 months of age	1.42	(1.28–1.58)			
After 6 months of age	ref				
**Daycare center during first 6 month** ^3^			0.0062	-	0.13
No daycare center attendance	ref				
Some daycare center attendance	1.66	(1.16–2.38)			

* Bonferroni corrected p-value estimated from the value for trend

** To few individuals, to estimate an adjusted OR

° Data from the statistics Denmark.

^1^ Data from first telephone interview at gestational week 12.

^2^ Data from the second telephone interview at gestational week 30.

^3^ Data from the third interview at 6 month post partum.

Postnatal factors associated with increased risk of OM were living together with older siblings and terminating breastfeeding before the age of six months. The number of siblings was associated with a 3-fold increased risk of early OM. Early daycare attendance was defined as attending a day care center, with more than five children attending the same center. Early daycare attendance was not significantly associated with early OM, when the Bonferroni correction was introduced. Socioeconomic status did not affect the risk of early OM when adjusted for the rest of the risk variables (Table 3). In a test of strength and potential bias between responders and non-responders differences, it was found that birthweight, gestational age at birth, parity, socioeconomic status, single parenthood, maternal smoking and maternal alcohol consumption during pregnancy, all were unequally distributed. Among non-responders, more women were smoking between 1 and 10 cigarettes/day (67% versus. 11% in responders), and more women were smoking more than 10 cigarettes/day (25% versus 5% in responders). Among responders, the frequency of women living without another adult in the household, were higher that among non-responders (2% versus 0%) (Table 4).

**Table 4:** Prevalence and characteristics of responders and non-responders in the DNBC.

Analysis of strength	Completed interviews (n =)	(%)	Not completed interviews (n =)	(%)	P-value
**Gender** °					0.4491
Girl	33803	48.95	5679	48.57	
Boy	35259	51.05	6014	51.43	
**Birthweight** °					<0.0001
Very low birth weight (< 1500 g)	44	0.07	39	0.34	
Low birth weight (1500 g—2499 g)	1241	1.85	260	2.29	
Normal birth weight (2500 g—4000 g)	51224	76.25	8767	77.2	
Weight = > 4000 grams	14674	21.84	2290	20.17	
**Gestational age at birth** ^2^					<0.0001
Preterm (Before 38th gestational week)	66231	95.84	11074	94.62	
Term (in or after 38th gestational week)	2874	4.16	630	5.38	
**Parity** °					<0.0001
1	32439	46.94	5569	47.58	
2	25507	36.91	4473	38.22	
3	9128	13.21	1378	11.77	
4+	2028	2.93	284	2.43	
**Socioeconomic status** °					<0.0001
1	13710	20.7	2640	23.82	
2	21278	32.13	3556	32.08	
3	25273	38.17	3905	35.23	
4	4652	7.03	780	7.04	
5	1306	1.97	203	1.83	
**Maternal age at birth** °					0.2926
Age < 20	310	0.45	54	0.46	
20 < = age < 30	32467	47.33	5498	47.26	
30 < = age <40	35175	51.28	5993	51.52	
40 < = age	643	0.94	88	0.76	
**Single parenthood** ^2^					<0.0001
No	67544	97.84	11683	99.95	
Yes	1493	2.16	6	0.05	
**Maternal smoking during pregnancy** ^1^					<0.0001
Non-smoking	54613	82.71	115	6.27	
Between 1 and 10 cigarettes a day	7803	11.82	1244	67.87	
More than 10 cigarettes a day	3611	5.47	474	25.86	
**Maternal alcohol during pregnancy** * ^1^					0.0025
No alcohol	45038	68.97	7370	67.61	
Yes. 2 or less units a week	15981	24.47	2731	25.06	
Yes. more than 2 units a week	4286	6.56	799	7.33	

* Alcohol consummation is calculated as the sum of maternal reported beers, wine and spirits consumed every week.

° Data from the statistics Denmark.

^1^ Data from the second telephone interview at gestational week 30.

^2^ Data from the third interview at 6 month post partum.

## Discussion

In line with the objectives, this study investigated the effect of a variety of risk exposures for OM at six months of age. The study showed that risk of maternal reported early OM was increased in children born by women who had taken penicillin during pregnancy. Other prenatal factors as male gender, young maternal age, low maternal self-estimated health and low gestational age at birth was also associated with increased risk of early OM. After birth, the most important factors associated with increased risk of early OM before the age of six months in the adjusted multiple logistic model were having siblings and early termination of breastfeeding.

### Limitations

During follow-up 11,683 eligible mothers and their children were non-responders. Because decision on not to participate in the follow-up was made by the woman, on behalf of the child, characteristics of non-responders reflect maternal characteristics. The test for strength of the results pointed at this tendency. Women not attending follow-up were more prone to be smoking. In addition, child characteristics did not seem to be associated with not attending follow-up, see Table 4.

Additional analyses to assess the effect of missing data on exposure variables were not performed. Although we assume that the information on exposure were “missing completely at random” or “missing at random” and imputation would have been possible, we did not replace the missing data[38]. Furthermore, the present study did not assess interactions between variables, although interactions between exposure variables might have affected the associations found.

A major limitation in this study was the use of parental reported disease outcome. This limitation is described later in more detail.

### Strengths

Analyses were based on 69,105 children. This is more than most other studies investigating diet and environmental effect on risk of early OM. Prior studies have been criticized for not sufficiently adjusting for relevant social factors[30,31]. The large size cohort with detailed information on maternal factors and information on life during pregnancy made it possible to include a wide range of socioeconomic confounding variables. Additionally, the present study included 14 relevant confounders to separate the effect of different exposure behaviors.

#### Bias

The present study was susceptible to different sorts of potential bias.

The data were collected by telephone, and the aim of the study was unknown to the interviewers. The cohort was a multipurpose cohort, and the outcome was therefore independent of the interviewer and the interviewer’s health beliefs. Therefore, the study was unlikely to suffer from interviewer bias.

During enrollment, only Danish speaking women were eligible for inclusion, because participants needed to have some basic Danish language skills. Excluding immigrant women not capable of speaking Danish potentially introduced selection bias, due to exclusion of a subpopulation prone to marginalization and unemployment. A study by Jacobsen et al. concerning bias from non-responders in the DNBC, reported an underrepresentation in the DNBC by non-academic women, women not working as health professionals and from women being unemployed, single, on high social supports and below the age of 20[39]. Many of these characteristics might be associated with risk of early OM. This could subvert a possible effect of these variables on the risk of early OM and render an impact of the generalizability of our findings[40].

#### Imprecision

A possible imprecision on both our outcome and exposure measures arise from recall bias. Items in the two prenatal interviews assessed exposure in the time interval prior to the interviews at 12 weeks and between 12th and 30th gestational week. Exposure measures during pregnancy were believed to be less susceptible to recall bias, as most pregnant women are very much aware of exposures during this period. However, the present prospective cohort design may be vulnerable to bias from differentiated recall of exposures between Women in different socioeconomic or educational strata. We believe that such exposure recall bias generally diminish an existing relation between the exposure being measured and early OM[41]. The outcome measure obtained 30 weeks after birth is more vulnerable to differentiated recall differences between groups because of the longer time period the women have to recall. This could potentially influence the result both positively or negatively. However, we do not believe that the outcome measure is prone to this bias. Todberg et al. explored the internal validity of the items regarding reported episodes of OM and found a high internal correlation between reporting episodes of OM at six month after birth, and reporting OM episodes at 18 month after birth[6]. This supports a statement of only minor influence of bias from recall on our results.

#### Outcome measure

Data used in this study was exclusively questionnaire data and primarily of qualitative nature. The outcome measure of maternal-reported OM has been validated in other studies[42–46]. Maternal-reported OM was not specified into acute OM, OM with effusion or ROM. Therefore, OM in this study covers the whole spectrum of different middle ear infections and inflammatory diseases.

As in other studies using parent-reported disease measures, this study was affected by imprecision in the outcome information due to mismatch between the true clinical diagnosis and maternal disease reports. Kvestad et al.[46] reported, that inconsistency between parent-reported disease and otological findings, were related to less severe disease. In contrast, additional questions regarding disease incidence and treatment increased accuracy of parent-reported OM[42]. In a study by Alho et al.[43], the parent-reported cumulative incidence between children younger than two years of age, was lower than the incidence found in medical records. Thus, the true incidence of OM can be expected to be even higher than reported in medical records as not all OM episodes results in a medical contact. Therefore, we expect that maternal reported episodes of early OM is an underestimate of the true incidence.

Because information about exposure was collected before birth, the imprecision on early OM reports were considered equally distributed among exposed and non-exposed. Although it was possible that women with good health insight would tend to report the numbers of OM closer to the actual numbers of episodes compared to women without the same health insight. In addition, women with health insight would tend to demonstrate a low-risk behavior during pregnancy. This would draw the risk estimation towards the null.

#### Exposure measures

The exposure measures were afflicted with the same imprecision as the outcome measures in form of possible misclassification bias, recall bias and detection bias which could have underestimated subclinical or borderline exposure measures or risk-behavior. In addition, each exposure variable might have its own individual mechanisms of imprecision and bias.

Reports on medicine consumption might be affected by misclassification. Not only were the reports subject to recall bias, but imprecision also increased due to low pharmacological compliance. A study by Olesen et al., estimated the compliance of used antibiotics in the DNBC at 52% [47]. This misclassification might also draw the risk estimation of the effect of penicillin treatment during pregnancy on early age OM towards the null hypothesis of no effect. Furthermore, the exposure for penicillin was afflicted with confounding by indication, as an indication for prescription precede penicillin consumption, thus the effect of penicillin during pregnancy on child’s early OM experience could be a reflection of the true effect from an unknown causal factor.

### Comparison

Only a few studies have assessed risk exposure during pregnancy related to early life OM. The overall prevalence of OM before six months of age was estimated to be 5.3% and thus in the lower end compared to a prevalence between 2% and 20% found by Engel and colleagues[42]. Another study that assessed early onset OM in a high-risk population of American Indians found that 63% of the children had experienced at least one episode of OM before the age of six months. This study however, did not use maternal reported OM, but instead clinical findings[15]. Finally, Daly and colleagues estimated the percentage of children who had suffered from episodes of either AOM or OME at age six months in an American birth cohort of 596 participants at 48%[48].

The individual risk factor estimates also differ from some existing publications. The elevated risk found for male children is in line with some of the literature[27,49], although the majority of published studies have been unable to find any association between early age OM and gender[11,15,28,48,50,51].

Furthermore, this study’s findings of peri- and postnatal risk exposures from low birthweight, preterm birth, high parity and early termination of breastfeeding support findings in other studies. Having siblings seems to constitute a considerable risk of acquiring early age OM, and demonstrates a dose-response relationship. In the study by Daly et al. the relative risk (RR) of AOM at six months was 1.2 having one sibling compared to no siblings. The RR was 1.4 having two or more siblings in the household versus no siblings[14]. Also risk modification of early termination of breastfeeding resemble results from earlier studies[11,52].

Smoking during pregnancy and early OM have been carefully examined in previous studies. We did not include smoking during pregnancy in our final model, as the effect of smoking was closely correlated with other social variables, and the independent effect of smoking during pregnancy became insignificant, when other socioeconomic variables were included in the final model. However, our crude risk estimate of OR equal to 1.23 (95% confidence interval 1.12–1.37) for smoking less than 10 cigarettes/day, is in accordance with results from Haaberg and colleagues who reported a 1.27 crude OR for children having experienced one or more reported episodes of acute OM before the age of six months[53]. Lieu et al. estimated an adjusted OR of the child having OM before six month of age at 1.08 (95% confidence interval 1.02–1.14) if the woman had been smoking during pregnancy[7]. However, a meta-analysis of six studies on prenatal smoking exposure, found no significant relation between maternal smoking during pregnancy and middle ear diseases in general[29].

Alcohol consumption was not associated with an increase in reported early OM in our study. This finding is in line with other studies on effect of alcohol during pregnancy and early OM[15]. In a study from 2014, Brennan-Jones et al. found an increased OR of parent-reported ROM at three years after birth in children of women drinking alcohol during pregnancy[28]. However, when controlling for social variables in the study, the risk became insignificant.

Maternal use of penicillin during pregnancy and risk of OM in offspring have to our knowledge not been studied before. We found an OR of 1.35 in risk of early OM (Table 3). Whether this finding is due to penicillin use itself or an underlying infection during pregnancy cannot be determined from this study.

### Generalizability

Although this study reflects an era before introduction of the pneumococcal vaccination in 2007 in Denmark, children before the age of six months are not fully protected by the vaccine[54–56]. Hence, these results can be extrapolated to most western societies and health systems. Still many risk exposures may differ between rural and urban populations, and may not be applicable in a homogenous subpopulation with different characteristics compared to the present studied population.

## Conclusion

The present study evaluated already known risk factors together with new potential factors not previously examined in relation to early OM. We did not in this large study find prenatal factors that could be used as predictors for the risk of early OM in childhood. However, we found a number of maternal characteristics as maternal age at birth, maternal self-estimated health and early life variables as low gestational age, high parity, and length of breastfeeding that predicts a higher risk of early OM. This knowledge may be important to parents and physicians in their speculations of the reasons why some children attracts early OM.
