# Correction: Risk Factors of Early Otitis Media in the Danish National Birth Cohort

**DOI:** 10.1371/journal.pone.0171901

**Published:** 2017-02-06

**Authors:** Asbjørn Kørvel-Hanquist, Anders Koch, Janni Niclasen, Jesper Dammeyer, Jørgen Lous, Sjurdur Frodi Olsen, Preben Homøe

The fourth author’s name is spelled incorrectly. The correct name is: Jesper Dammeyer. The correct citation is: Kørvel-Hanquist A, Koch A, Niclasen J, Dammeyer J, Lous J, Olsen SF, et al. (2016) Risk Factors of Early Otitis Media in the Danish National Birth Cohort. PLoS ONE 11(11): e0166465. doi:10.1371/journal.pone.0166465
